# Gut microbiota, nutrition, and mental health

**DOI:** 10.3389/fnut.2024.1337889

**Published:** 2024-02-09

**Authors:** Gia Merlo, Gabrielle Bachtel, Steven G. Sugden

**Affiliations:** ^1^Department of Psychiatry, New York University Grossman School of Medicine and Rory Meyers College of Nursing, New York, NY, United States; ^2^Lake Erie College of Osteopathic Medicine, Erie, PA, United States; ^3^Department of Psychiatry, The University of Utah School of Medicine, Salt Lake City, UT, United States

**Keywords:** brain-gut-microbiota axis, neuroplasticity, mental health, brain health, nutritional psychiatry, lifestyle psychiatry

## Abstract

The human brain remains one of the greatest challenges for modern medicine, yet it is one of the most integral and sometimes overlooked aspects of medicine. The human brain consists of roughly 100 billion neurons, 100 trillion neuronal connections and consumes about 20–25% of the body’s energy. Emerging evidence highlights that insufficient or inadequate nutrition is linked to an increased risk of brain health, mental health, and psychological functioning compromise. A core component of this relationship includes the intricate dynamics of the brain-gut-microbiota (BGM) system, which is a progressively recognized factor in the sphere of mental/brain health. The bidirectional relationship between the brain, gut, and gut microbiota along the BGM system not only affects nutrient absorption and utilization, but also it exerts substantial influence on cognitive processes, mood regulation, neuroplasticity, and other indices of mental/brain health. Neuroplasticity is the brain’s capacity for adaptation and neural regeneration in response to stimuli. Understanding neuroplasticity and considering interventions that enhance the remarkable ability of the brain to change through experience constitutes a burgeoning area of research that has substantial potential for improving well-being, resilience, and overall brain health through optimal nutrition and lifestyle interventions. The nexus of lifestyle interventions and both academic and clinical perspectives of nutritional neuroscience emerges as a potent tool to enhance patient outcomes, proactively mitigate mental/brain health challenges, and improve the management and treatment of existing mental/brain health conditions by championing health-promoting dietary patterns, rectifying nutritional deficiencies, and seamlessly integrating nutrition-centered strategies into clinical care.

## 1 Introduction

The complex interplay between the foods we eat and how our brains react to nutrition highlights the interconnectedness of daily lifestyle habits and the health of the brain, mind, and body. Understanding the relationship between food and its impact on mental/brain health, in conjunction with the reciprocal influence of mental/brain health on whole-body health, including gut health, and daily lifestyle habits, such as dietary choices, requires a transdisciplinary approach that incorporates the fields of psychiatry, psychology, neuroscience, nutrition, and lifestyle medicine (1–4). This review will explore the significant impact of nutrition on brain health, mental well-being, and cognitive functioning. It will highlight the emerging role of lifestyle interventions and nutritional neuroscience in proactively improving patient outcomes and managing mental/brain health conditions.

The brain has the greatest metabolic demands (approximately 20–25% of the body’s total energy consumption) of any human organ (5). Additionally, optimal brain health requires various nutrients, including carbohydrates, essential fatty acids, proteins, vitamins, and minerals (6, 7). Glucose derived from carbohydrates is the primary source of energy for the brain. Essential fatty acids (e.g., omega-3 fatty acids, omega-6 fatty acids) play a critical role in maintaining the integrity of structures in the brain, as well as promoting the synthesis and functioning of neurotransmitters and components of the immune system (8). Finally, amino acids found in protein foods, including tryptophan, tyrosine, histidine, and arginine, are also utilized by the brain to produce neurotransmitters and neuromodulatory compounds (9). Research surrounding the link between nutrition and mental/brain health issues has transitioned from an initial focus on nutrient deficiencies that manifested observable psychiatric and/or neurological symptoms in clinical settings (i.e., the lack of thiamine and the potential development of Wernicke syndrome) to a prioritization of research inquiries aiming to better understand the implications of comprehensive dietary patterns on human well-being in the context of both health and disease (4).

Evidence in the burgeoning fields of lifestyle psychiatry and nutritional neuroscience has made it increasingly evident that diet is not only a matter of physical sustenance but also a fundamental factor influencing cognitive abilities, emotional states, and risk of or protection against mental health issues and/or brain illnesses (4, 10–13). This bidirectional relationship challenges the historical silos of nutrition and neuroscience, emphasizing the need for transdisciplinary collaboration to unravel the intricacies of how the foods we consume shape the functioning of our brains and, in turn, how the state of our brains influences our dietary choices.

## 2 Intersection of nutrition and mental health

The intersection between nutrition and mental health is an emerging area of research interest. For the purpose of this paper, we will focus on depression since the data is more complete and depression is projected to be one of the top health concerns by 2030 (14). Despite growing medications and therapeutics, the negative impact of depression continues to grow as reflected by the increase in disability-adjusted life-years (DALYs), years lived with disability (YLDs), and years of life lost (YLLs) (15).

### 2.1 Five theories of neuropathology of depression

In their 2016 review, Loonen and Ivanona postulated five theories regarding the neuropathology of depression (16). First is the well known monoamine theory. The majority of psychotropic medications that have FDA approval for the treatment of depression (e.g., selective serotonin reuptake inhibitors, serotonin-norepinephrine reuptake inhibitors, or tricyclic acids) modulate serotonin, as such, the monoamine theory links the origins of depression to deficits of serotonin. Currently, this has been expanded to include norepinephrine and dopamine as well. Second is the biorhythm theory, which centers on sleep disruption and altercations within the REM sleep and deep sleep patterns. A dysfunctional sleep rhythm alters the natural circadian rhythm that is regulated within the nucleus suprachiasmaticus (SCN), It is hypothesized that the altered circadian rhythm within the SCN contributes to mood dysregulation patterns throughout the day.

Third is the neuro-endocrine hypothesis. Thyroid levels, particularly, hypothyroidism has been linked with the onset of depressive symptoms (17). Changes in thyroid hormones have been linked with serotonin insufficiency (18). As will be discussed in more detail, dysregulation within the HPA, particularly higher levels of cortisol have been linked with altercations of the circadian rhythm, hippocampus, and limbic system, which may account for changes of emotions seen in depression. Individuals with depression also as a whole have higher levels of cortisol in the mornings and evenings compared to non-depressed individuals (19). The fourth is the neuro-immune hypothesis, which also will be discussed in more detail. The HPA axis and the immune system are significantly interconnected, and changes with cortisol levels can greatly impact the immune system. Changes within neurohormones or other triggers cause the release of neurotrophic factors, like BDNF, regulatory cytokines, etc., which have been linked with changes in the hippocampus, limbic system, SCN and have been linked with depression. Finally, the fifth is the kindling hypothesis, which states that the actual illness causes cell death and reinforces depressive symptoms with progressive worsening symptoms.

In the 2021 review, Marx et al., show dietary patterns, commonly known as the Western Diet or Standard American Diet, which are high in saturated fats, refined carbohydrates and ultra-processed foods, can create many of the Loonen and Ivanona hypotheses (16, 20). A systematic analysis of the Global Burden of Disease Study assessing the health effects of dietary risks in 195 countries from 1990 to 2017 found that diet-related risk factors were responsible for approximately one-fourth of all deaths among adults and almost one-fifth of all disability-adjusted life years among adults (21). Particularly concerning is the increasing consumption of UPF. Prior to COVID-19 pandemic, the typical U.S. diet consisted of about 60% UPF. The consumption patterns were consistent between gender and race (22), and the global consumption of UPF has increased in the post-pandemic era (23). UPF contains excessively high levels of refined sugars, saturated fat, trans-fat, caffeine, and sodium, with regards to both overall macro- and micronutrient content and energy density, as well as very low levels of dietary fiber.

### 2.2 Ultra-processed food and neuropathology

In an attempt to categorize food content, the NOVA food classification system was developed, which categorizes food into four subgroups: 1) unprocessed and minimally processed foods, 2) processed culinary ingredients, 3) processed foods, and 4) ultra-processed food (UPF), which are defined as “food substances of no or rare culinary use” (24, 25). A 2021 meta-analysis from Lane et al. of over 345,000 individuals noted that higher rates of UPF predicted an increased risk of subsequent mental health symptoms (26). In a cross-section design of 10,359 participants, Hecht et al. showed that individuals who consumed primarily NOVA4, had an odds ratio for developing depression (OR: 1.81; 95% CI 1.09, 3.02). They had a risk ratio for being more mentally unhealthy (RR: 1.22; 95% CI 1.18, 1.25). Finally, they were also significantly less likely to report zero mentally unhealthy days (OR: 0.60; 95% CI 0.41, 0.88) (27). A potential explanation may be how a diet rich in UPF leads to dysregulated neuroimmune responses (28), increased neuroinflammation (29, 30), and altercations within the neuroendocrine system (29).

### 2.3 Ultra-processed food and the gut

People suffering from mental illness consume more UPF (30) and people who consume more UPF are prone to develop mental illness (31, 32). Apart from the neuroinflammatory contribution of mental illness on the microbiota, diets high in UPF promote low-grade inflammation, affecting the microbiota (29). In a recent meta-analysis conducted by Nikolova et al. (33), 34 studies comprising more than 1,500 individuals with mental health illness were evaluated and showed a pattern of microbiota among mental illness diagnoses, which were depleted of pyruvate-producing bacteria. It is important to note that this shift in microbial diversity, which affects the absorption of key amino acids and adversely affects brain health (34, 35).

The following sections delve into the current state of understanding regarding these intersections, shedding light on the mechanisms through which nutrition influences mental well-being and offering insights into potential avenues for therapeutic interventions.

## 3 The brain-gut-microbiota: the developmental origins and a review

### 3.1 Developmental origins

The BGM system is a bidirectional communication network connecting the gastrointestinal system and the brain that has emerged as a focal point linking nutrition, health, and well-being (36, 37), particularly related to mental/brain health. The gut microbiota is a diverse community of microorganisms inhabiting the digestive tract that influences various aspects of brain function through the production of neurotransmitters, immune signaling molecules, and metabolic substances (38). Dietary patterns alter the microbiota composition of the gut (39). Strengthening the gut microbiota through dietary interventions holds promise as a novel approach to ameliorate mental/brain health symptoms, risk factors, and protective factors as the BGM system is a primary mechanistic channel through which the gut microbiota exert their effects on mental/brain health through nutrition-related pathways (4, 33).

The gut and brain are both derived from neural crest tissue during embryogenesis and influence each other during human developmental processes as they integrate into the enteric nervous system (40, 41). Microbes initially colonize the gastrointestinal tract at the time of birth. A microbiota is a biological community that forms when microorganisms live in a specific habitat and produce genetic material. The human microbiota develops after the first year of life and continues to diversify throughout life. Evidence indicates that microbiota bacteria have undergone a co-evolutionary process alongside humans and engage in bidirectional physiological interactions with our bodily systems (42). It is estimated that the ratio of microbial cells to human cells in the body is approximately one-to-one, which points to the significance of microbiota in facilitating processes that support human flourishing across the lifespan. There are three primary pathways through which the gut microbiota interacts with the brain along the BGM system, including neuroimmune, neuroendocrine, and vagus nerve pathways (37).

### 3.2 BGM system-neuroimmune pathway

Dietary habits play a huge role in maintaining a healthy gut microbiota (3), which inturn plays a key role in modulating the immune system. Diets rich in dietary fibers, not UPFs, activate microbial enzymes within certain bacteria (*Bifidobacterium, Lactobacillus, Lachnospiraceae, Blautia, Coprococcus, Roseburia, and Faecalibacterium*) are able to break down complex carbohydrates (43), via a fermentation, into short-chain fatty acids (SCFA), namely acetate, propionate, and butyrate (3). These SCFA have a wide range of host activities, including metabolism, cell differentiation, gene regulation (3, 44), and regulating anti-inflammatory and pro-inflammatory cytokines (45). Within the gut, SCFAs strengthen the epithelial barrier functions, which maintains an favorable environment for commensal bacteria and inhibits pathogen’s growth (44).

Butyrate is metabolized into acetyl CoA, which is needed in mitochondrial metabolism. It also plays a pivotal role in regulating IL-10 receptors and maintaining the gut epithelial tight junctions (44). Without this, a change of the overall gut microbiota diversity can develop, which may lead to a condition known as dysbiosis. The growth of *Enterobacteriaceae*, especially *Escherichia, Shigella, Proteus,* and *Klebsiella* can increase in enterotoxin levels (46), which leads to dysbiosis. This pro-inflammatory microbial imbalance typically occurs in diets rich in UPFs, sodium, saturated fats, *trans*-fat, and refined sugar (47, 48). It is significant to note that dysbiosis can lead to dysregulated immune responses that can contribute to chronic inflammation and have a wide range of negative health implications, including health challenges such as neuropsychiatric conditions and autoimmune diseases in which neuroinflammation is a key contributing factor (49, 50).

Specifically, chronic inflammation from the *Enterobacteriaceae* can release lipopolysaccharide (LPS) from their own cells. Once released within the gut, they impair gut-associated lymphoid tissue (GALT), which includes the multi-follicular Peyer’s patches of the ileum, the numerous isolated lymphoid follicles (ILF) distributed along the length of the intestine, and the vermiform appendix (28). Additionally, the disruption of the immune system within the enteric system will alter system immunity. Additional critical consequences of LPS include: increases blood brain barrier permeability, altering the microglia of the CNS as it promotes gliosis and neuronal damage (51). Due to the breakdown of the blood brain barrier, there is an increase of plasma proteins into the brain, particularly the component proteins, which can adversely affect synaptic pruning (52).

Communication between the gut microbiota and the immune system occurs as part of the broader BGM system, rather than in isolation. Bidirectional crosstalk between these two major biological systems happens in such a way that signals from the gut can affect the brain and vice versa. The immune system may send signals to the brain when it detects inflammation in the gut, which can affect mood, behavior, and cognitive function. There is evidence that this bidirectional communication can contribute to the development or progression of neuropsychiatric conditions such as depression, anxiety, and as well as other mood disorders (47).

### 3.3 BGM system-neuroendocrine pathway

The neuroendocrine pathway of the BGM system involves a complex network of communication between the brain, the gut, and the endocrine system (37). In addition, the production of LPS has been shown to activate the hypothalamic–pituitary–adrenal axis (HPA) (53). Heightened levels of cortisol can in turn change intestinal permeability by activating interferon gamma, interleukin 6, interleukin 1 beta, and tumor necrosis factor alpha (54), which alter the diversity of the gut microbiota, leading to dysbiosis (55), neuronal damage within microglia and astrocytes, and depletion of neurotrophic factors, like BDNF (56). Heightened levels of cortisol can affect brain function by decreasing prefrontal cortex activity, heightening amygdala fear response and decreasing functional memory due to its negative hippocampal interactions.

Additionally, there is a growing body of literature that a number of neurotransmitters that function as hormones are also mediated within the BGM axis. Serotonin, for example, is derived from the essential amino acid tryptophan, which is absorbed within the kynurenine pathway (34) and regulated through the gut microbiota (46, 55). Serotonin concentration within the brain can contribute to the development and or progression of depression and anxiety (57). *Enterobacteriaceae* have also been shown to be histamine producing bacteria (58). Histamine has been linked with visceral gut hypersensitivity, increased gut permeability and altered gut motility (59), and studies have linked depression to these heightened eosinophilic conditions (60).

### 3.4 BGM system-vagus nerve

The vagal pathway of the BGM system involves the vagus nerve, the tenth cranial nerve, that plays a significant role in the bidirectional transmission of signals between the brain and the gastrointestinal tract. The vagus nerve extends from the brainstem into the abdomen through the gastrointestinal tract and other organs, including the heart and lungs and transmits both sensory and motor signals throughout the mesenteric organs. The sensory components of the vagus nerve called vagal afferents relay information from numerous organs to the brain, including signals associated with the gut environment such as the presence of inflammation, gut distension, nutrient availability, and gut hormones (i.e., leptin, ghrelin, glucagon-like peptide 1, and insulin), and gut microbial metabolites (29, 61). Bidirectional communication between the brain and gut relies on an axis composed of 80% afferent and 20% efferent neurons (62). These microbial sensory signals can convey information about the composition and activity of the gut microbiota to the brain. The vagus nerve, which is also composed of motor branches known as vagal efferents, sends signals from the brain to the gastrointestinal tract and other organs. Vagal motor signals can influence functions of the gastrointestinal tract like gut motility, the secretion of digestive enzymes, and the modulation of immune responses in GALT (63).

## 4 Brain health, mental health, and wellness

While mental health is known by both the public and scientific community, it does not capture the importance of the concept of *brain health*. Brain health is defined by the WHO as the fostering of optimal brain development, cognitive health, and well-being throughout the entire lifespan (64). As such, brain health includes what is thought, done, said, and felt. The lifespan continuum of brain health is also in alignment with current evidence regarding neuroplasticity, the ability of the brain to adapt and escape previous genomic restrictions through the formation and reorganization of synaptic connections.

**Figure 1:**
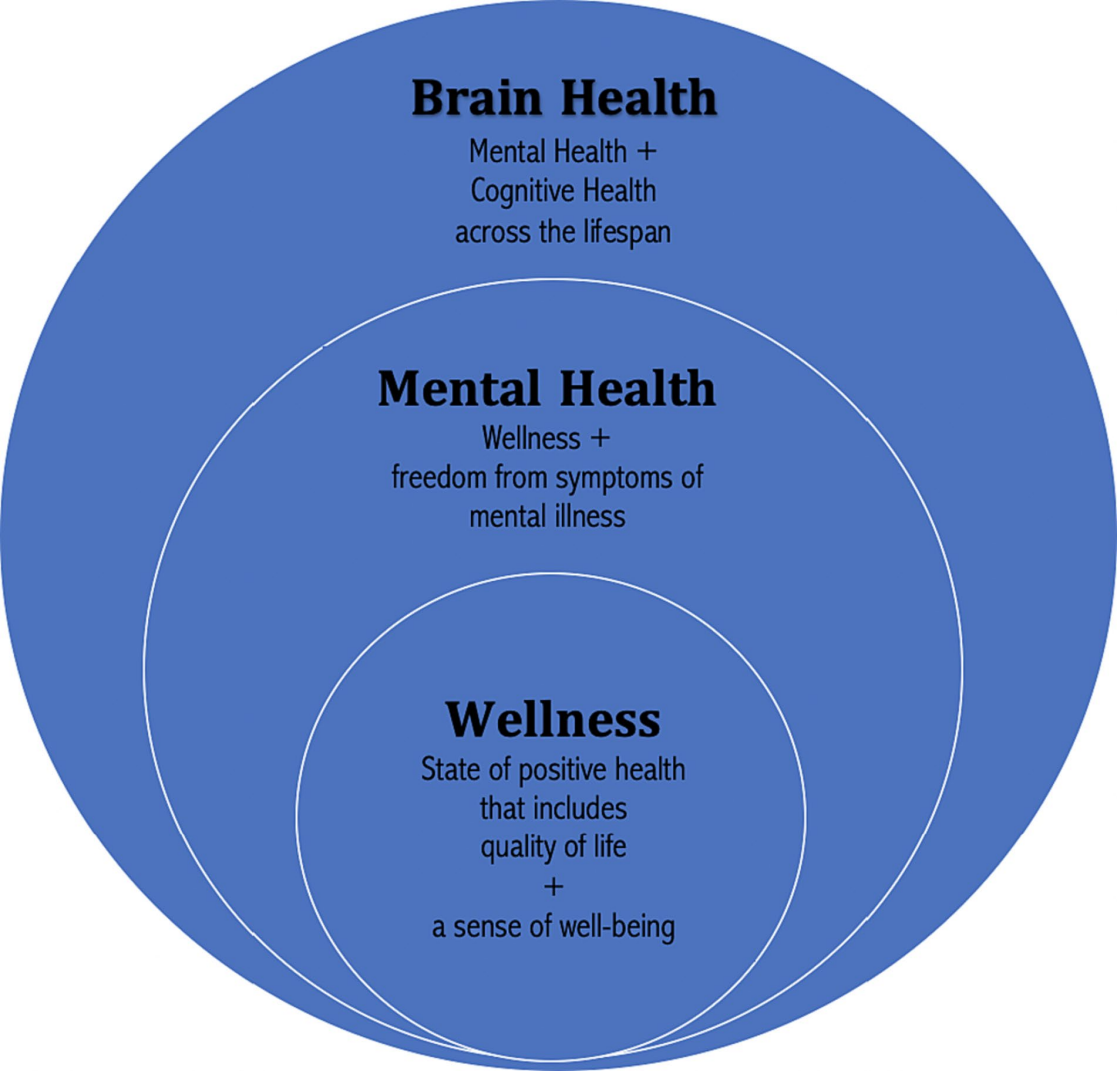
Relationship between wellness, mental health, and brain health. Copyright 2023. From Lifestyle Psychiatry by Gia Merlo & Chris Fagundes. Reproduced by permission of Taylor and Francis Group, LLC, a division of Informa plc.

Brain health includes the concepts of wellness, mental health, and brain health (65) (see Figure 1 for a representation of the relationship between wellness, mental health, and brain health). With this framework in mind, it is important to note how dietary choices across the lifespan have the potential to positively alter brain structure and improve brain health or negatively impact it.

## 5 Nutrition and brain changes across the lifespan

The brain and body are exposed to a wide array of exogenous and endogenous stimuli that can impart various levels of functionality across biopsychosocial spheres of health, which determine both short- and long-term outcomes across the lifespan. A healthy early life development of the BGM system helps prevent later disease development. Nutritional programming is the concept that key cells within the body will be able to absorb nutrients or synthesize them *de novo* in key periods of time to support early life development (66). Multiple nutrients with epigenetic potential that are present in the diet or produced via microbial metabolism in the human gut. The B-complex vitamins, SCFAs, and polyphenols are among nutrients known to exert epigenetic effects on the host (67). Significant synaptogenesis in the brain occurs simultaneous to diversification of gut bacteria during critical windows (68, 69). Critical windows are periods of development during which the phenotypic outcomes (e.g., intelligence quotient) of an individual are remarkably sensitive to interactions between environmental and genetic factors. Environmental factors, such as diet, can significantly alter the developmental course of high-plasticity bodily systems, such as the central nervous system, cardiovascular system, gastrointestinal system, and immune system (70). As such, within the nutritional framework, nutrients need to be available for optimal development (66).

### 5.1 Fetal period

The fetal period is a key nexus period within the nutritional framework. It is estimated that over half of the maternal energy available to the growing fetus during pregnancy is allocated to brain development (71). The brain health, quality of life, and overall well-being of offspring are critically dependent on the trajectory of their neurodevelopment, which begins prenatally (72, 73). An explanation for the relationship between postnatal neurodevelopment and gut microbiota diversity may lie in their influence on each other’s maturation processes mediated through the BGM axis (74).

Diet plays a critical role in the development of the gut microbiota in early life (75, 76). Research has demonstrated that the presence of breast- or formula-feeding, as well as the timing of transition to solid foods, are major drivers in shifting the gut microbiota to a more adult-like composition (77). Gut microbiota composition varies between infants fed breast milk versus formula. The microbiota of breast-fed infants primarily consists of *Bifidobacterium*, *Lactobacillus*, and *Staphylococcus* (77, 78). Notably, the microbiota of formula-fed infants is predominantly *Clostridium*, *Anaerostipes*, and *Roseburia*. Moreover, data indicates that the introduction of solid foods around 6 months of age (within the critical neurodevelopmental window) in infants with an immature gut microbiota can fortify their health by stimulating the growth of microbes typically present in the adult gut (79). Adult-like gut microbiota are typically inclusive of *Anaerostipes*, *Bacteroides*, *Bilophila*, *Clostridium*, and *Roseburia* (77).

It has also been noted that breast milk feeding and duration of breastfeeding are positively associated with enhanced structural connectivity of neuronal networks in both white and gray matter, including regions of the brain consistent with improved outcomes related to cognitive and behavioral performance (80–83). Studies have not only revealed that levels of cognitive function of breast-fed children are significantly higher than formula-fed children at six to twenty-three months of age, but also that this distinction in brain performance persists over time as children age (84–86).

### 5.2 Early life

Early life eating patterns, which modulate and prime the gut microbiota have been found to influence both short- and long-term human health and disease, including brain health and heart disease (87). Codagnone et al. (88) identified the gut microbiota as the fourth most significant element in the programming of brain health and disease during early life, in addition to host genetics and prenatal and postnatal environment. Adolescence is a critical period of neurodevelopment that coincides with maturing socially, cognitively, and improving executive function (89). Typical adolescent diets tend to be high in UPF, high in sugar containing foods and beverages, and low in fiber. Adolescent diets can contribute to mental health symptoms, yet experts are still investigating the crossroads of a healthy gut microbiota, a developing brain, and brain health (90).

### 5.3 Adult life

Composition of gut microbiota gradually shifts throughout the lifespan and has been shown to play a role in the regulation of age-related changes in cognitive function and immunity (91). It is of significance to note that the current global burden of disease is mainly caused by neuropsychiatric conditions and cardiovascular disease. Poor diet, which can induce dysbiosis, has been established as one of the major contributing factors to the global burden of disease (92). Individuals affected by neuropsychiatric conditions are also more likely to be affected by chronic conditions, such as cardiovascular diseases (93). Daily lifestyle choices, including healthy or unhealthy eating patterns, can serve as protective factors or risk factors for the development and progression of neuropsychiatric conditions and chronic health conditions.

Individual differences in lifestyle-related factors, such as diet, contribute to cognitive reserve and physical reserve. Cognitive reserve is the brain’s adaptive capacity to be resilient against neural damage or age-related cognitive decline by efficiently using available brain resources through pre-existing or compensatory processes (94). Physical reserve is the body’s capacity to maintain physical functionality in the setting of illness, injury, or age-related physiological changes (95). Robust evidence indicates healthy eating (96). The World Health Organization describes a healthy diet as an eating pattern that is rich in fruits, vegetables, legumes, nuts, and whole grains and restricted in refined sugar, salt, saturated fats, and *trans*-fats (97). A healthy diet has been found to be positively correlated with cognitive reserve and cognitive function (98).

### 5.4 Elderly life

The gut microbiota composition again changes in this phase of adult life as the bacterial diversity decreases. Opportunistic bacteria, like the *Enterobacteriaceae* family, increase in density (99). Animal models have shown that higher concentrations of LPS in adults can lead to increased blood brain permeability, tau hyperphosphorylation and additional neuroinflammation and may account for cognitive deficits (100). In a cross-sectional study of 127 participants, Saji et al. found a correlation between LPS concentration and mild cognitive impairment (odds ratio: 2.09, 95% confidence interval: 1.14–3.84, *p* = 0.007) (101).

## 6 BGM system and neuroplasticity

Brain development is a process that occurs throughout the lifespan of the individual. After the initial fetal development, which is driven by the actual genetics of the fetus, subsequent development, also called neuroplasticity, is significantly driven by environmental and epigenetic changes. Changes can provide benefits or maladaptive effects that can lead to a spectrum of neuropsychiatric sequelae (102). In a recent review, Innocenti identifies five potential targets of neuroplastic change: 1) neuronal cell count, 2) neuronal cell migration, 3) differentiation of the somatodendritic and axonal phenotypes, 4) formation of neuronal connections or pathways, and 5) cytoarchitectonic differentiation within the microglia. The intent of this section is not to provide a definitive review of all possible implications but rather show examples within the metabolic, immune, neuronal, and endocrine pathways.

### 6.1 Neuronal cell count

Neurogenesis is a fundamental component of neuroplasticity, as well as the functional and structural processes related to brain health homeostasis. The formation of new neuronal cells continues in the adult hippocampus throughout the course of the lifespan. The hippocampus is a region in the brain that modulates learning, memory, and mood and is extremely sensitive to environmental stimuli, including diet (103). Evidence suggests that decreased neurogenesis contributes to cognitive impairment and neuropsychological conditions, such as anxiety and depression (104, 105). Zainuddin & Thuret suggest that nutrition can influence adult hippocampal neurogenesis via four primary pathways, including caloric intake, the nutritional content of meals, frequency of meals, and texture of meals (106).

### 6.2 Neuronal cell migration

Recent literature suggests that neuropsychiatric disorders and cognitive dysfunction are linked to damage induced by oxidative stress on nuclear and mitochondrial DNA (107, 108). Oxidative stress is a key pathway through which dietary factors affect neuronal cell migration. Of note, reactive oxygen species (ROS) involved in pathways related to oxidative stress have been found to induce epigenetic changes in aging processes of the brain (109), as oxidative damage has been recorded as a significant contributor to neurodegeneration (110). Increased production of free radical production and inflammatory responses may occur when long-term exposure to prooxidant and proinflammatory metabolic factors exceeds the capacity of bodily systems to protect against tissue injury and mitochondrial damage (111). Long-standing excess in certain nutritional components can induce a metabolic state of chronic, dysregulated, low-level inflammation often co-occurs with mitochondrial dysfunction (112).

Oxidative stress and damage can be caused by prooxidant foods, which have been found to decrease antioxidant activity and increase the production of ROS (113). The quality and quantity of various macronutrients and micronutrients can interact with prooxidant or antioxidant metabolic pathways (114). Data indicates that oxidative mechanisms inhibit the proliferation of precursor neurons, as well as the migration, differentiation, and viability of new neuronal cells (115, 116).

### 6.3 Cell phenotypes

The differentiation process of a neuron into somato-dendritic or axonal phenotypes engenders alterations in metabolic needs that vary from those of precursor or stem neuronal cells and are mediated by dietary nutrients (117). Differentiated mature neurons require higher levels of energy. The increased energy needs of the developing or mature brain is bolstered through a metabolic shift from energy primarily sourced from cytoplasmic aerobic glycolysis to neuronal oxidative phosphorylation modulated by the mitochondria (117, 118). It is important to note that the reprogramming of metabolic processes related to the oxidation, glycolysis, and mitochondria supports neurogenesis and the functionality of differentiated neuronal cells by promoting adequate epigenetic modulation of gene expression and increased signaling of neurotransmitters in both the central nervous system and across the BGM system.

### 6.4 Neuronal connections

Increasing evidence substantiates that the both cognitive and non-cognitive functions of the brain encoded in the human genome are vulnerable to modification via epigenetic and epitranscriptomic mechanisms (119–122). The bidirectional relationship between food and the BGM system can alter neuroplasticity through interactions with these epigenetic and epitranscriptomic pathways. Nutrient intake can regulate gene expression that modulates learning, memory, and adaptive behaviors (123). Alternatively, neuroplastic changes in learning, memory, and adaptive behaviors can alter gene expression to promote different eating patterns. For instance, peptide hormones, such as ghrelin, leptin, and insulin, which are implicated in physiological processes surrounding hunger and satiety, utilize nutrient sensing along the BGM system to modulate signals in the brain related to hunger, satiety, and food-induced reward (124). Reception of these signals then shapes adaptive behaviors and experiential learning and conditioning associated with food intake, which can regulate mechanisms for the activation or repression of certain genes (125).

### 6.5 Cytoarchitectonic differentiation

Bioactive nutritional compounds may have the potential to restore quiescent capacity of microglia, which are cells that play a vital role in neurodevelopment (e.g., neuronal proliferation, neuronal differentiation), brain health homeostasis, neuroplasticity, and injury response and repair mechanisms in the central nervous system. Dysregulation of microglial cytoarchitectonic differentiation has implications in neuroinflammation, cognitive deficits, neuropsychiatric conditions, and chronic inflammatory diseases (126). Cytoarchitectonic differentiation within the microglia denotes the process of area-specific structural organization of these nervous system-specific macrophage cells. Neuroinflammatory responses can be induced by microglial cells in response to immune signals from the periphery. Evidence has demonstrated that age-related alterations in the brain cause changes in microglial morphology, as well as attenuated microglial functional capability to regulate injury and repair processes through adequate homeostatic shifts between anti-inflammatory and proinflammatory states and migratory and clearance abilities (127). Epigenetic modifications also change microglial functional profiles (128). Data suggests that microglia exposed to different environmental factors early in life, such as certain nutrients, may have implications in microglial diversity in later life through these epigenetic modifications (129).

## 7 Neuropsychiatric disorders affected by food

As previously discussed, there is a bidirectional link between metabolic aberrations and a diverse array of neuropsychiatric disorders (130–132). Approximately one in three individuals with a chronic health condition are affected by co-occurring neuropsychiatric condition (133, 134). Not surprisingly, the neuropsychiatric conditions most affected by dietary factors, anxiety and depression (135), are also those most prevalent in the general population. Diet-related pathways for therapeutic targets include the BGM system, inflammation, oxidative stress, mitochondrial dysfunction, epigenetics, the HPA axis, and tryptophan-kynurenine metabolism (20), which address many of neural circuits implicated in depression (16).

As noted, depression continues to be one of the world’s most debilitating illnesses. World estimates have estimated the lifetime prevalence between 30 and 40% (136). It is not uncommon for symptoms to last at least 1 year in duration (137). Depression is comorbid with other neuropsychiatric illnesses (i.e., anxiety, cognitive impairment, post-traumatic stress disorder, substance use disorders) and chronic physical disorders (i.e., arthritis, asthma, cancer, cardiovascular disease, chronic pain, chronic respiratory disorders, diabetes, hypertension, obesity) (138). Those with these comorbid outcomes tend to have worse outcomes (137). As a result, individuals with depressive disorders commonly develop a pattern of dysbiosis, showing higher concentrations of the *Bacteroides* species, a species that alters the BGM system (139, 140).

Historically, the treatment of choice for depression has been the use of medications that modulate the uptake of serotonin (i.e., selective serotonin reuptake inhibitors). Many individuals, however, have not received symptom relief from these remedies (141). Lessale et al., conducted a meta analysis of 21 cross sectional studies and 20 longitudinal studies. Individuals were either adhering to the Mediterranean diet (MD), the Healthy Eating Index, the Alternative Healthy Eating Index, the Dietary Approaches to Stop Hypertension, or the Dietary Inflammatory Index. Individuals on the MD had an estimated relative risk of developing depression of 0.67 (5% CI 0.55–0.82) (142). Firth et al. examined 16 randomized clinical controlled trials (dietary interventions varied) comprising over 45,000 participants and noted that dietary interventions improved depressive symptoms compared to controls (g = 0.275, 95% CI = 0.10 to 0.45, *p* = 0.002) (29). The SMILES study, a randomized control study, had study participants avoid UPF and fast food. The study showed that the number needed to improve depressive symptoms was 4.1 (143). A meta-analysis from 2018 of epidemiological studies showed that every increase in 100 g of whole fruits or vegetables corresponded with a 5% reduction risk of depression (144). Given these findings the Royal Australian and New Zealand College of Psychiatrists recommend lifestyle changes, including diet and exercise, and therapy for mild and moderate forms of depression (145). Similarly, the World Federation of Society for Biological Psychiatry has adopted a WFBP within its treatment recommendations for depression (146).

## 8 Future directions related to the gut microbiota, nutrition, and mental health

Prior to the COVID-19 pandemic, it was estimated that 50% of the United States experienced loneliness (147). Following the pandemic, the impact of loneliness has increased. More adolescents report loneliness and isolation and chronic illness. Although there is not a direct correlation between isolation, chronic illness and mental health, those who experience isolation report having increasing mental health symptoms (148). Based on world events, social injustice, and worsening socioeconomic factors, it seems unlikely that these trends will reverse. Although it may seem trivial in the wake of these factors to focus on the BGM system and nutrition, this effort may have more of a global impact. In addition to the mental health symptoms as outlined, dietary choices are the largest driver of chronic illness (21). The health industry promotes protein as the optimal food ingredient to reverse these trends and overlooks the importance of fiber (43). As a result, less than 5% of Americans are consuming an adequate dose of fiber (149), which has been key in developing and maintaining a healthy BGM-system (3, 34, 150).

Traditionally, healthcare professionals receive very little evidence-based nutrition didactics during their formal education (151). The reasons vary from nutrition being a “soft science,” or there is “too much controversy over the nutrition literature,” or there is “only time to teach what is on licensing exams.” To ensure that healthcare providers receive this vital training, licensure boards need to incorporate questions regarding plant-based nutrition and the importance of the BGM system. Additionally, we encourage other medical organizations to follow the Royal Australian and New Zealand College of Psychiatrists and World Federation of Society for Biological Psychiatry and adopt lifestyle guidelines within their mental recommendations.

## Author contributions

GM: Writing – original draft, Writing – review & editing. GB: Writing – original draft, Writing – review & editing. SS: Writing – original draft, Writing – review & editing.
